# Increased Skeletal Muscle 11βHSD1 mRNA Is Associated with Lower Muscle Strength in Ageing

**DOI:** 10.1371/journal.pone.0084057

**Published:** 2013-12-31

**Authors:** Alixe H. M. Kilgour, Iain J. Gallagher, Alasdair M. J. MacLullich, Ruth Andrew, Calum D. Gray, Philippa Hyde, Henning Wackerhage, Holger Husi, James A. Ross, John M. Starr, Karen E. Chapman, Kenneth C. H. Fearon, Brian R. Walker, Carolyn A. Greig

**Affiliations:** 1 Centre for Cognitive Ageing and Cognitive Epidemiology, Geriatric Medicine Unit, University of Edinburgh, Edinburgh, United Kingdom; 2 Department of Clinical and Surgical Sciences, Division of Health Sciences, School of Clinical Sciences, University of Edinburgh, Edinburgh, United Kingdom; 3 Endocrinology Unit, Centre for Cardiovascular Science, Queen’s Medical Research Institute, University of Edinburgh, Edinburgh, United Kingdom; 4 Clinical Research Imaging Centre, Queen’s Medical Research Institute, University of Edinburgh, Edinburgh, United Kingdom; 5 School of Medical Sciences, University of Aberdeen, Aberdeen, United Kingdom; INSERM/UMR 1048, France

## Abstract

**Background:**

Sarcopenia, the loss of muscle mass and function with age, is associated with increased morbidity and mortality. Current understanding of the underlying mechanisms is limited. Glucocorticoids (GC) in excess cause muscle weakness and atrophy. We hypothesized that GC may contribute to sarcopenia through elevated circulating levels or increased glucocorticoid receptor (GR) signaling by increased expression of either GR or the GC-amplifying enzyme 11beta-hydroxysteroid dehydrogenase type 1 (11βHSD1) in muscle.

**Methods:**

There were 82 participants; group 1 comprised 33 older men (mean age 70.2years, SD 4.4) and 19 younger men (22.2years, 1.7) and group 2 comprised 16 older men (79.1years, 3.4) and 14 older women (80.1years, 3.7). We measured muscle strength, mid-thigh cross-sectional area, fasting morning plasma cortisol, quadriceps muscle GR and 11βHSD1 mRNA, and urinary glucocorticoid metabolites. Data were analysed using multiple linear regression adjusting for age, gender and body size.

**Results:**

Muscle strength and size were not associated with plasma cortisol, total urinary glucocorticoids or the ratio of urinary 5β-tetrahydrocortisol +5α-tetrahydrocortisol to tetrahydrocortisone (an index of systemic 11βHSD activity). Muscle strength was associated with 11βHSD1 mRNA levels (β -0.35, p = 0.04), but GR mRNA levels were not significantly associated with muscle strength or size.

**Conclusion:**

Although circulating levels of GC are not associated with muscle strength or size in either gender, increased cortisol generation within muscle by 11βHSD1 may contribute to loss of muscle strength with age, a key component of sarcopenia. Inhibition of 11βHSD1 may have therapeutic potential in sarcopenia.

## Introduction

Sarcopenia is the loss of muscle mass and function which accompanies even healthy ageing [1]–[3]. Both muscle mass and function (i.e., power and strength) begin to decline from the third decade with mass reducing by 1–2% per year and strength reducing by around 2% per year [4]–[8]. Sarcopenia is associated with an increased risk of falls and fractures, disability, loss of independence and mortality [9]–[11]. Despite this public health problem, current understanding of the mechanisms underlying sarcopenia is limited, hampering progress in the development of novel treatments for maintenance of muscle mass and physical independence in old age. Theories underlying the development of sarcopenia include: inflammation, cellular senescence, hormones and growth factors and lifestyle factors (eg nutrition) [12], [13].

One possible mechanism within the field of hormones and growth factors is glucocorticoid dysregulation. It is well known that glucocorticoids at pharmacological levels or in spontaneous Cushing’s syndrome cause myopathy, with a combination of muscle atrophy and dysfunction. Glucocorticoids are believed to effect these changes on muscle through a combination of increased protein breakdown (particularly through the ubiquitin-proteasome system) [14], decreased protein synthesis (by inhibiting transport of amino acids into muscle and inhibiting the action of insulin and IGF-1) [15] and decreasing production of IGF-1 and myostatin [14]. In the context of sarcopenia, this mechanism could occur via elevated circulating glucocorticoids due to age-related hypothalamic-pituitary-adrenal (HPA) axis dysregulation. Alternatively, it could occur selectively within the muscle, by increased activity of the glucocorticoid receptor (GR) or the enzyme 11β-hydroxysteroid dehydrogenase type 1 (11βHSD1). 11βHSD1 converts inactive cortisone to active cortisol and is known to be present and biologically active in human muscle as well as many other tissues [16]–[18]. Indeed a recent study by Tiganescu et al found that elevated 11βHSD1 activity was inceased in skin biopsies from older adults compared to younger adults and that this increased activity was associated with markers of skin ageing (eg dermal atrophy and deranged collagen structural organization) [19]. Establishing links between GC and sarcopenia could lead to novel therapies, as several 11βHSD1 inhibitors are currently in clinical development for type 2 diabetes and other degenerative diseases, including cognitive dysfunction [20].

There is some evidence of an association between increased plasma and salivary cortisol and lower muscle mass and strength but these data are inconsistent [21]–[24]. Glucocorticoid metabolites in a 24 hour urine sample may be more informative than plasma cortisol levels since they reflect glucocorticoid status over the diurnal cycle. Additionally ratios of the metabolites can be used as an index of peripheral 11βHSD activity [25]. However, no studies to date have examined the relationship between urinary glucocorticoid metabolites and sarcopenia. Similarly, there are no published data examining the relationship between GR and 11βHSD1 expression and muscle loss and function in older adults. Importantly, expression of 11βHSD1 and GR mRNA has been shown to reflect glucocorticoid activity, for example there is a correlation between 11βHSD1 mRNA expression and enzyme function [26].

The aim of this study was to investigate the relationship between plasma and urinary glucocorticoid metabolites and levels of mRNA encoding GR and 11βHSD1 in skeletal muscle, with muscle size and strength. We hypothesized that increased glucocorticoid signaling in skeletal muscle acting through GR by, (a) elevated circulating cortisol, (b) increased expression of 11βHSD1 or (c) increased expression of GR, is associated with reduced muscle size and strength.

## Methods

### Participants

Participants were healthy volunteers recruited at two sites in Scotland: young and older men were recruited in Aberdeen (Group 1) and older men and women were recruited in Edinburgh (Group 2). This allowed us to test for possible age and gender effects. Participants were defined as healthy after applying previously published health selection criteria to the responses to a questionnaire [27]. Existing samples were available from two nearby cities in Scotland so these were used for analysis, rather than beginning a new de novo cohort collection. No comparisons were made between these two independent cohorts.

### Ethics Statement

Written informed consent was obtained and all procedures received local ethical committee approval. In Edinburgh this was by the Lothian Local Research Ethics Committee 02 and in Aberdeen this was by the North of Scotland Research Ethics Committees. The study conformed to the standards set by the Declaration of Helsinki.

### Anthropometry

Body weight was measured with participants in light clothing using a beam scale (Seca, UK). Height was measured using a wall mounted stadiometer.

### Muscle Function

Maximum voluntary isometric knee extensor strength was measured using an established method [28]. Following instruction, the participant made a maximum voluntary contraction (Newtons) which was held for 5 seconds. Three separate measurements were obtained and the highest value was used in subsequent analysis.

### Muscle Size

Mid-thigh quadriceps cross-sectional area (CSA) was measured using a 1.5T MR scanner (Phillips Gyroscan Intera). T1-weighted axial images were taken with the isocentre of the magnetic field located at the mid-femur point which was landmarked prior to the scan according to International Standards of Anthropometric Assessment (ISAK) guidelines 2001. Imaging parameters were: slice thickness 10 mm; acquisition matrix 512×512; echo time (TE) 15 ms; repetition time (TR) 425 ms; and flip angle 90°. The CSA of the quadriceps was quantified using Analyze 8.0 (Mayo Clinic, Rochester, USA) according to a previously published technique [29]. Two of the subjects from Group 2 did not undergo MRI due to claustrophobic symptoms.

### Plasma Cortisol

Blood samples were obtained from participants in the morning after overnight fast (mean time 0945h, range 0915–1030h). Plasma cortisol was measured by competitive immunoassay with direct chemiluminescent technology using the Bayer Advia Centaur method (see http://labmed.ucsf.edu/labmanual/db/resource/Centaur_Cortisol.pdf).

### Quadriceps Muscle Biopsy

Quadriceps femoris samples were obtained from the region of vastus lateralis via percutaneous needle biopsy using a Bergstrom needle [30]. The biopsy was obtained in a sterile environment by sharp dissection under local anaesthetic using 1% lidocaine. The samples were then snap frozen in liquid nitrogen and stored at −80°C before analysis [31].

### RNA Isolation

Total RNA was isolated from quadriceps muscle biopsies using the Qiazol reagent (Qiagen, Crawley, UK) and miRNeasy RNA isolation columns (Qiagen, Crawley, UK). Briefly biopsies were homogenised in 1400 ul or 700 ul Qiazol depending on the size of the tissue sample using a Polytron PT1200E (Kinematica AG). Total RNA was isolated from the homogenised muscle using miRNEasy columns with an on column DNAse treatment step using the RNase-Free DNase Set (Qiagen, Crawley, UK). After elution from the column into 30 ul nuclease free H_2_O, RNA was quantified using the Nanodrop instrument (Labtech, UK) and quality assessed using the Bioanalyzer (Agilent, UK). All samples had 260/280 ratios above 1.8, and RIN scores above 7.5.

### cDNA Preparation and qPCR

RNA samples were converted to cDNA using the Ovation RNA Amplification kit (Nugen, Netherlands). RNA was diluted to 10 ng/ul and 50 ng total RNA was used in the amplification reaction carried out according to the manufacturer’s instructions, yielding between 3 ug and 11 ug cDNA. For qPCR, cDNA was diluted to ∼50 ng/ul. Quantitative RT-PCR reactions were run, in triplicate, on an Applied Biosystems Step One Plus system. The reaction mix was POWER SYBR Green x2 Master mix 12.5 ul, forward primer (10 uM) 1 ul, reverse primer (10 uM) 1 ul, H2O 9.5 ul and cDNA 1 ul. Reaction conditions were 95°C for 10 mins, 95°C for 15s, 60°C for 60s (40 cycles) followed by melting curve generation from 60°C to 95°C. Ct values were examined and within triplicates any value greater than 0.3 Ct were removed before means were calculated. Data were then analysed using the delta Ct method with HPRT as a normaliser. After normalisation data were inverted and scaled such that the largest value for each gene was set to 100.

Primer sequences used were NR3C1 FP – CTGTCGCTTCTCAATCAGACTC; RP – GCATTGCTTACTGAGCCTTTTG; 11βHSD1 FP – AGGCTGCTGCCTGCTTAGGA; RP – AGCCCCAGAATGGGGAGGAGA; HPRT FP – TGACACTGGCAAAACAATGCA; RP- GGTCCTTTTCACCAGCAAGCT. HPRT was chosen as a normaliser as preliminary analysis of housekeeping gene performance showed HPRT to be stable across samples and expressed at a similar level to genes of interest compared to b-actin, GAPDH, b2M and 18S.

### Urinary Glucocorticoid Metabolism

24 hour urine samples were collected to quantify urinary glucocorticoid metabolites using gas chromatography electron impact mass spectrometry following solid phase extraction, hydrolysis of conjugates and formation of their methoxime-trimethylsilyl derivatives, as described previously [25].

Two composites of the data were used in subsequent analyses. Firstly, total urinary steroids, comprising the sum of 5β-tetrahydrocortisol (5βTHF), 5α-tetrahydrocortisol (5αTHF), the main urinary metabolites of cortisol, and tetrahydrocortisone (THE), the main urinary metabolite of cortisone (total urinary GC = 5βTHF +5αTHF+THE). Secondly, an indirect indicator of systemic 11βHSD activity, comprising the ratio of 5βTHF and 5αTHF to THE (ratio of cortisol to cortisone metabolites = (5βTHF +5αTHF)/THE).

### Statistical Analysis

Statistical analysis was performed using SPSS version 18.0. Bivariate correlations were performed using Spearman’s rho to allow analysis of the non-parametric variables. Forced entry multiple linear regression was performed and the data from the two groups were analysed separately, when possible, to test reproducibility. Due to the large difference in age between the older and younger groups, age was analysed as a binary variable in the multivariate regression. Group 1 (n = 52) had 80% power at the p = 0.05 level to detect a correlation of r = 0.38 and Group 2 (n = 30) had 80% power at the p = 0.05 level to detect a correlation of r = 0.49. In view of the power calculations and the exploratory nature of the study, adjusting for multiple hypotheses testing was not deemed to be appropriate.

## Results

In total, 82 participants were recruited. Table 1 shows numbers of participants and their age, height, BMI, and the main outcome variables for each group. Independent t tests found significant sex and age related differences for height, muscle size and muscle strength but not for BMI or any measure of glucocorticoid status (Table 1). Table 2 shows bivariate correlations, which confirmed that measures of body size (height and BMI) were significantly associated with muscle size and strength. Therefore in constructing multivariate models we adjusted for body size as well as age and gender, which had been selected *a priori* due to their accepted relationships with muscle size and strength. BMI correlated with total urinary GC (rho = 0.60, p = 0.0005) and plasma cortisol (rho = −0.52, p = 0.006), whereas height did not significantly correlate with total urinary GC or plasma cortisol (p>0.05 for both). Therefore in multivariate analyses with urinary GC and plasma cortisol as predictor variables we adjusted for potential confounding by BMI, gender and age (see Tables 3 & 4). Neither BMI nor height correlated with the muscle GR or 11βHSD1 mRNA expression levels. Therefore because height correlated more significantly with muscle size and strength than BMI (Table 2), we adjusted for height and gender for the multivariate analyses with muscle GR and 11βHSD1 mRNA as predictor variables (Table 4).

**Table 1:** Group characteristics.

	Group 1 youngermen (n = 19)	Group 1 oldermen (n = 33)	p-value^a^	Group 2 oldermen (n = 16)	Group 2 olderwomen (n = 14)	p-value^b^
**Age (years)**	22.2 (1.7)	70.2 (4.4)	<0.001	79.1 (3.4)	80.1 (3.7)	n/s
**Height (cm)**	177.6 (6.7)	171.9 (5.4)	0.001	171.3 (6.1)	157.6 (5.9)	<0.001
**BMI (kg/m^2^)**	24.0 (2.5)	25.2 (2.5)	n/s	25.3 (3.9)	24.1 (3.1)	n/s
**Muscle Size (cm^2^)**	92.7 (11.5)	67.3 (7.4)	<0.001	63.5 (7.3)	43.8 (6.8)	<0.001
**Muscle Strength (Newton)**	774.9 (136.6)	525.2 (73.6)	<0.001	364.7 (79.7)	273.4 (73.4)	0.003
Total Urinary GC* (microg/day)	9887 (7721–18372)	10224 (7841–17000)	n/s	8192 (5534–12506)	4925 (3699–6806)	n/s
**11βHSD activity (urine THFs:THE)**	1.12 (0.37)	1.15 (0.45)	n/s	1.28 (0.79)	0.81 (0.49)	n/s
**Plasma cortisol (nmol/litre)**	–	–		349 (106)	321 (65)	n/s
**GR mRNA**	–	–		59.4 (24.5)	58.3 (18.8)	n/s
**11βHSD1 mRNA**	–	–		25.3 (19.7)	32.2 (31.8)	n/s

Data are mean (SD) except *non-parametric data therefore median and IQ range shown.

a. Independent t test between younger and older men in Group 1.

b. Independent t test between men and women in Group 2.

n/s = not significant.

**Table 2:** Bivariate correlations including muscle size and strength.

	Group 1 muscle size	Group 1 muscle strength	Group 2 muscle size	Group 2 muscle strength
**Height**	.34*	.22	.68**	.42*
**BMI**	–.04	–.07	.32	.38*
**Total urinary GC**	–.04	.01	.61**	.45*
**11βHSD activity**	–.09	–.05	.35	.16
**Plasma cortisol**	–	–	–.20	–.31
**GR mRNA**	–	–	.11	.04
**11βHSD1 mRNA**	–	–	–.15	–.29

Data are Spearman’s Rho Correlation Coefficients.

p<0.01 (2-tailed).

p<0.05 (2-tailed).

**Table 3:** Regression coefficients for the glucocorticoid measures in models predicting muscle size/strength (Group 1).

Glucorticoid Measure	Muscle Size *Beta (sig, n)*	Muscle Strength *Beta (sig, n)*
**Total Urinary GC** a	−0.10 (p>0.05, 52)	<−0.01 (p>0.05, 52)
**THFs:THE** a	<0.01 (p>0.05, 52)	0.04 (p>0.05, 52)

^a^ adjusting for age and BMI.

**Table 4:** Regression coefficients for the glucocorticoid measures in models predicting muscle size/strength (Group 2).

Glucorticoid Measure	Muscle Size *Beta (sig, n)*	Muscle Strength *Beta (sig, n)*
**Plasma Cortisol** a	−0.12 (p>0.05, 25)	−0.35 (p>0.05, 27)
**Total Urinary GC** a	0.23 (p>0.05, 28)	0.18 (p>0.05, 30)
**THFs:THE** a	0.08 (p>0.05, 28)	0.10 (p>0.05, 30)
**GR mRNA** b	0.03 (p>0.05, 20)	0.04 (p>0.05, 22)
**11βHSD1 mRNA** b	−0.17 (p>0.05, 20)	−0.35 (p = 0.04, 22)

^a^ adjusting for gender and BMI.

^b^ adjusting for gender and height.

Plasma cortisol was measured in Group 2 only. There were no significant association between fasting morning plasma cortisol and muscle size and a non-significant negative trend with muscle strength (β −0.35, p = 0.08) (Table 4). In both groups neither total urinary glucocorticoids nor the ratio of cortisol:cortisone metabolites were associated with muscle size or strength (Tables 3 & 4).

We used muscle biopsies from a subset of Group 2 to examine the relationships between GR and 11βHSD1 mRNA levels and muscle size and strength. Increased 11βHSD1 mRNA was significantly associated with lower muscle strength after adjustment for sex and height (β −0.35, p = 0.039, n = 22∶12 men mean age 79.8 (sd 3.6) and 10 women, mean age 80.5 (sd 4.1)). There were no significant relationships between GR mRNA and muscle size or strength, or between 11βHSD1 mRNA and muscle size (Table 4).

## Discussion

This study investigated the relationship between circulating and tissue indices of glucocorticoid status and muscle size and strength in two groups. Group 1 allowed comparison of older with younger men. There were no age differences in urinary cortisol metabolites, although muscle biopsies were not obtained in this group so we did not test the effect of ageing *per se* on muscle mRNA levels. Group 2 allowed comparison of older men with older women. There were no differences in plasma cortisol, urinary glucocorticoid metabolites or muscle GR or 11βHSD1 mRNA levels between the sexes in this relatively small sample. Within each group we explored associations between glucocorticoid variables and muscle size and strength after adjustment for potential confounding effects of age, gender and body size as appropriate. In these analyses, indices of HPA axis function, including morning plasma cortisol and 24 h urinary cortisol metabolite excretion, were not associated with muscle strength or size. Additionally urinary cortisol:cortisone metabolite ratios, which principally reflect 11βHSD activity in the major organs of liver and kidney, were not associated with muscle strength or size. However, in muscle itself, higher levels of mRNA encoding the cortisol-amplifying enzyme 11βHSD1 were associated with reduced muscle strength. This finding is consistent with the hypothesis that enhanced glucocorticoid signalling within muscle contributes to sarcopenia.

To our knowledge there have been no previous investigations of muscle glucocorticoid signaling in human sarcopenia. We hypothesized that because 11βHSD1 and GR regulate the exposure of target tissues to glucocorticoids, increased expression of 11βHSD1 and GR could therefore contribute to sarcopenia in the absence of an increase in circulating GCs. We found that increased 11βHSD1 mRNA expression in muscle is associated with lower muscle strength. This is consistent with this hypothesis. We did not find a relationship between 11βHSD1 mRNA expression and muscle size, but in normal ageing, muscle strength is reported to deteriorate more rapidly than muscle size; suggesting a decline in force generating capacity with age [5], [32]. A number of contributory mechanisms have been proposed to explain this (eg increased muscle fibre stiffness); our data suggest a possible role for increased GC action at the muscle level. GC may affect strength more than muscle mass by exacerbating glycation of the myosin molecule, which appears to slow the intrinsic shortening velocity of the muscle fibre, decrease force per cross-sectional area and increase intramuscular collagen cross-linking which can cause muscle stiffness [33], [34]. In addition, GC may cause mitochondrial dysfunction and reduced oxidative capacity, which would similarly result in a decrease in force generating capacity [35]. 11βHSD1 is known to act locally within muscle, resulting in measurable production of cortisol in samples from veins draining human muscle, and therefore increased 11βHSD1 mRNA expression is likely to increase myocellular cortisol levels thereby mediating these effects [18]. More research with larger samples and with a wider range of severity of sarcopenia is required to investigate the relationship between 11βHSD1 expression and activity and muscle ageing.

We found no relationship between GR mRNA expression and muscle mass or strength. It is possible that polymorphisms of GR modulate the effect of GC on muscle, and that level of expression is less important than genotype. For example male carriers of the ER22/23EK polymorphism in GR, which is associated with relative GC resistance, have greater muscle mass and strength than non-carriers [36].

There are no published studies investigating the association between urinary GC and muscle size or strength. However, there are studies reporting associations between salivary and plasma GCs and muscle size and function. In a previous study of men and women >75 years higher salivary, but not serum, cortisol was associated with lower appendicular skeletal mass (ASM) measured using DEXA [21]. Similarly, in a large longitudinal ageing study higher salivary but not serum cortisol predicted loss of grip strength over 6 years, but there was no association of cortisol with baseline grip strength or ASM [22]. A smaller study including both young and older men found that increased serum cortisol correlated with lower knee extensor strength in both age groups and with quadriceps cross-sectional area only in the older group [23]. The Caerphilly Prospective Study, which included measurements of cortisol status and physical performance over 20 years, found that higher mid-life plasma cortisol predicted faster walking speeds in older age, although salivary cortisol did not correlate with walking speed or balance in older age [24]. Collectively, these studies provide contradictory evidence relating salivary or plasma cortisol to muscle strength and mass. Taken with our data, there does not appear to be a consistent association between activation of the HPA axis and age-associated sarcopenia. These negative findings are important in excluding this plausible hypothesis.

Some limitations of this study should be acknowledged. We examined the effect of GC on ageing muscle using a younger and older group of volunteers separated in age by nearly 50 years and by many lifestyle factors; a problem inherent to cross-sectional studies. Longitudinal studies investigating rate of decline of muscle mass and function and measures of GC would be more informative but are difficult to conduct due to the slow decline of muscle mass and strength during ageing. The sample sizes were relatively modest, particularly with respect to muscle GC data which were obtained from only a subset of Group 2 who underwent muscle biopsy. It has also been shown that sarcopenia affects the upper and lower limbs differently and our study investigated only the lower limbs [4], [37]–[39]. Also, our healthy older volunteers constituted a sample which may be not fully representative of the ageing population; this may influence the generalisability of our results. Finally, we used mRNA expression as a marker of activity rather than a direct measure of 11βHSD1 activity, however several studies have found correlations between mRNA expression and enzyme activity in rodents and humans, so we regard mRNA as an appropriate indicator of 11β-HSD1 activity [26].

## Conclusion

Sarcopenia is one of the major causes of frailty and disability in older people. It is associated with greatly increased risk of loss of independence and institutionalization. In this novel investigation of healthy old and young people we found a significant association between increased muscle 11βHSD1 expression and lower quadriceps strength. We found no significant associations between plasma cortisol, urinary GC metabolites or GR expression and muscle mass or strength. Longitudinal studies are now required to investigate these relationships and to further explore the possibility of 11βHSD1 inhibitors as a novel treatment for sarcopenia.
